# Prevalence and correlates of contraceptive use among female adolescents in Ghana

**DOI:** 10.1186/s12905-015-0221-2

**Published:** 2015-08-19

**Authors:** Samuel H. Nyarko

**Affiliations:** Department of Population and Behavioural Sciences, School of Public Health, University of Health and Allied Sciences, Hohoe, V/R Ghana

## Abstract

**Background:**

Adolescence is a critical stage in the life course and evidence suggests that even though contraceptive use has been steadily increasing among women in Ghana over the past years, contraceptive prevalence and determinants among female adolescents is quite lacking. This paper examines the prevalence and correlates of contraceptive use among female adolescents in Ghana.

**Methods:**

The paper used data from the 2008 Ghana Demographic and Health survey. Bivariate analysis was carried out to determine the contraceptive prevalence among female adolescents while logistic regression analysis was applied to examine the correlates of female adolescent contraceptive use.

**Results:**

The study founded that female adolescent contraceptive use was significantly determined by age of adolescent, education, work status, knowledge of ovulatory cycle, visit of health facility and marital status.

**Conclusions:**

This has implications for adolescent sexual and reproductive health programmes in Ghana. It is therefore essential to intensify girl child education and strengthen the provision of family planning information and services for female adolescents in the country.

## Background

Preventing teenage pregnancy is considered a priority among policymakers and the public because of its high economic, social and health costs for teen parents and their families [1]. In spite of continuous global investments in adolescent sexual and reproductive health (ASRH) programmes, constraints such as limited knowledge and lack of access to resources as well as services still exist in effectively meeting the ASRH information and service needs of adolescents. These constraints are particularly evident in sub-Saharan Africa where unintended pregnancies and adolescent childbearing continue to be a burden [2–4]. In many parts of the region, adolescent sexuality and reproductive health remains a highly charged moral issue, which is compounded by the fact that in most cases, reproductive health services in the region are not oriented towards adequately meeting the needs of adolescents [5–7].

Female adolescents are unduly disadvantaged in terms of the risks of unintended pregnancies that are associated with poor outcomes such as miscarriages, stillbirths, unsafe abortion and other complications that might result in infant or maternal deaths [8, 9]. In developing countries, contraceptive use among young women, married or unmarried involves a lot of experimentation and is inconsistent [10]. Female adolescents face many barriers in the use of contraceptive methods which include fear, embarrassment, cost and lack of knowledge [10]. Consequently, most pregnancies to female adolescents in sub-Saharan Africa are unplanned and the use of contraceptive methods among this group remains low [11–13]. Thus, understanding the correlates of contraceptive use is quite vital to the efforts of promoting contraceptive use among female adolescents as well as the implementation of effective family planning programmes.

Family planning (contraceptive use) is particularly relevant to Millennium Development Goal (MDG) 5 of improving maternal health, which calls for the reduction of maternal mortality ratio by three quarters and the achievement of universal access to reproductive health by 2015. Evidence from some studies suggests that effective family planning programmes result in decreased maternal and infant morbidity as well as mortality [14–18]. It has been estimated that about one-third of maternal deaths and close to one-tenth of child mortality globally could be prevented annually with the help of family planning programmes [15]. Thus, by allowing women to postpone motherhood, space births and cease childbearing, contraceptive use reduces unwanted pregnancies and the demand for abortion [16, 19].

Previous studies have considered the determinants of contraceptive use in Ghana among women in the reproductive age group of 15 to 49 [20–23] and trends in contraceptive use among female adolescents in Ghana [24]. However, little is known about the prevalence and correlates of contraceptive use among female adolescents (aged 15 to 19) in Ghana. As such, this paper sought to examine the prevalence of contraceptive use and the correlates of contraceptive use among female adolescents in Ghana.

## Methods

Data for the study were extracted from the 2008 Ghana demographic and health survey (GDHS). The 2008 survey is the fifth survey in the series of national level population and health survey conducted as part of the global DHS programme. It is designed to provide data to monitor the population and health situation in Ghana as follow-on to the 1988, 1993, 1998 and 2003 GDHS surveys. It is a nationally representative survey comprising 4916 women aged 15–49 and 4568 men aged 15–59, that used a two-stage sample design for selecting households for the data collection. Data collection took place over a three-month period, from early September to late November, 2008 [25]. The dataset was requested from the Measure DHS program on April 1, 2011. An approval was then granted to download the data; therefore, no formal ethical approval was required in this case.

Data for women aged 15 to 19 were extracted from the dataset (*n* = 1037). The dependent (outcome) variable for the study is current contraceptive use including both traditional and modern methods. Current contraceptive use was dichotomous denoting users and non-users of contraceptive methods. The selected study variables include: age of adolescent, level of education, religious affiliation, ethnicity, marital status, work status (paid work), wealth status, knowledge of ovulatory cycle, use of health facility, place of residence and region of residence. Some of these study variables were re-coded to suit the purpose of the study while some were used as they are in the original dataset. For instance, religious affiliation was re-coded into Catholic, other Christian, Muslim and Traditional where “other Christians” refers to Christians apart from Catholics. Wealth status was also re-coded into poor, average and rich by combining “poorer” and “poor” for poor and “rich” and “richer” for rich.

The data were analysed in Stata 11 [26]. Bivariate analysis involving cross-tabulations was used to analyse contraceptive use prevalence and results presented in the form of proportions, while logistic regression analysis was applied to examine the correlates of female adolescent contraceptive use in Ghana. Odds ratios were also presented for significant variables in the model in order to estimate the likelihood of contraceptive use among the various categories of female adolescents. In order to cater for clustering, the data was weighted using an adjusted sample weight that was generated for the dataset.

## Results

### Prevalence of female adolescent contraceptive use

Table 1 presents the background characteristics and contraceptive prevalence among women aged 15 to 19 in Ghana. The overall contraceptive prevalence in the sample of 1037 women was 18.3 % comprising 14.6 % of modern methods and 3.7 % of traditional methods. As indicated in Table 1, 58.8 % of the female adolescents were aged 18 to 19 while 41.2 % were aged 15 to 17. The prevalence of contraceptive use was higher among female adolescents aged 18 to 19 (31.4 %) than female adolescents aged 15 to 17 (9.2 %). Also, 68.3 % had secondary or higher education, 23.3 % had primary education and 8.4 % had no formal education.

**Table 1:** Prevalence of contraceptive use among a sample of 1037 women in Ghana ages 15 to 19, by select characteristics: 2008

Characteristic	Frequency	Percent distribution	Percent using contraception
Age of woman			
15–17	610	58.8	9.2
18–19	427	41.2	31.4
Level of education			
No education	87	8.4	3.5
Primary	242	23.3	19.0
Secondary/Higher	708	68.3	19.9
Religious affiliation			
Catholic	170	16.4	14.7
Other Christian	623	60.0	20.1
Muslim	180	17.4	19.4
Traditional/spiritual	64	6.2	7.8
Ethnicity			
Akan	471	45.4	20.4
Ga/Dangme	60	5.8	30.0
Ewe	127	12.2	22.8
Mole-Dagbani	379	36.6	12.4
Marital status			
Not married	941	90.7	15.3
Married/living together	96	9.3	47.9
Work status			
Working	357	34.4	28.9
Not working	680	65.6	12.8
Wealth status			
Poor	436	42.0	15.4
Average	199	19.2	25.6
Rich	402	38.8	17.9
Knowledge of ovulatory cycle			
Yes	789	76.1	21.9
No	248	23.9	6.8
Visited health facility			
Yes	285	27.5	30.2
No	752	72.5	13.8
Place of residence			
Urban	447	43.1	21.0
Rural	590	56.9	16.3
Region of residence			
Western	95	9.1	15.9
Central	77	7.4	23.4
Greater Accra	130	12.5	21.5
Volta	89	8.6	18.0
Eastern	104	10.0	30.8
Ashanti	163	15.7	22.1
Brong Ahafo	76	7.3	19.7
Northern	109	10.5	4.6
Upper East	80	7.7	10.0
Upper west	114	11.0	14.9

Source: Computed from 2008 GDHS data set

In terms of contraceptive prevalence, the highest was found among female adolescents with secondary or higher education (19.9 %) while the lowest was among those without formal education (3.5 %). With regard to religious affiliation, 60 % were other Christians, 16.4 % were Catholics while 17.4 % were Muslims and only 6.4 % were Traditionalists/spiritualists. Contraceptive use was therefore highest among other Christians (20.1 %) and lowest among the traditionalists or spiritualists (7.8 %).

Most (45.4 %) of the respondents were Akans while only 5.8 % were Ga-Dangmes. Contraceptive use was however highest among the Ga-Dangmes (30.0 %) and lowest among the Mole-Dagbanis (12.4 %). Even though 90.7 % were not married while only 9.3 % were married/living together, contraceptive prevalence was higher among those who were married or living together (47.9 %) than among their counterparts who were unmarried (15.3 %). 

Also, 65.6 % were not working while 34.6 were working at the time of the survey. However, contraceptive prevalence was higher among the workers (28.9 %) than among the non-workers (12.8 %). In terms of wealth status, 42 % were from poor households while 19.2 % were from average households. Although most of them were from poor households, contraceptive use was lowest among those from poor households (15.4 %) and highest among those from average households (25.6 %).

The majority (76.1 %) had knowledge of their ovulatory cycle while 23.9 % had no knowledge of their ovulatory cycle. Contraceptive prevalence was found to be higher among those who had knowledge of their ovulatory cycle (21.9 %) than among their counterparts who had no knowledge of their ovulatory cycle (6.8 %). The majority (72.5 %) also had not visited any health facility while only 27.5 % had visited any health facility. Nevertheless, contraceptive prevalence was higher (30.2 %) among female adolescents who had visited health facility than those who had never visited any health facility (13.8 %).

Even though more than half (56.9 %) were from rural areas and 43.1 % were from urban areas, contraceptive prevalence was higher (21.0 %) among those from the urban areas than their counterparts from the rural areas (16.3 %). For regional variations, albeit more (15.7 %) female adolescents were from the Ashanti Region and the least (7.3 %) were from the Brong Ahafo Region, contraceptive prevalence was highest (30.8 %) among female adolescents from the Eastern Region and lowest among their counterparts from the Northern Region (4.6 %).

### Correlates of female adolescent contraceptive use

As indicated in Table 2, only six study variables had significant relationship with female adolescent contraceptive use. These include age of adolescent, level of education, work status, knowledge of ovulation cycle, visit of health facility and marital status. However, the study found no significant relationship between female adolescent contraceptive use and religious affiliation, ethnicity, wealth status, type of residence and region of residence. The odds of contraceptive use among female adolescents aged 18 to 19 were 3.49 times the odds of contraceptive use among those aged 15 to 17. Also, the odds of contraceptive use were 7.39 times among female adolescents who had primary education and 11.53 times among those who had secondary or higher education compared to their counterparts who had no formal education.

**Table 2:** Logistic regression of correlates of female adolescents’ contraceptive use

Variables	Odds ratio	*P*-value	95 % conf. interval
Age of adolescent			
15–17 (Ref)	1		
18–19	3.49***	0.000	2.32–4.15
Level of education			
No education (Ref)	1		
Primary	7.39**	0.003	1.88–2.72
Secondary/higher	11.53***	0.000	2.84–3.13
Religious affiliation			
Catholic (Ref)	1		
Other Christians	2.65	0.674	0.62–2.06
Muslim	1.14	0.064	1.21–3.78
Traditional/spiritual	1.05	0.942	0.32–3.36
Ethnicity			
Akan (Ref)	1		
Ga/Dangme	1.49	0.303	0.69–3.21
Ewe	1.48	0.258	0.75–2.97
Mole-Dagbani	0.79	0.509	0.38–1.59
Marital status			
Not married (Ref)	1		
Married/Living together	4.75***	0.000	2.48–4.86
Work status			
Not working (Ref)	1		
Working	2.99***	0.000	2.01–4.51
Wealth status			
Poor	1		
Average	1.37	0.274	0.77–2.41
Rich	1.65	0.172	0.35–1.20
Knowledge of ovulation cycle			
No	1		
Yes	2.89***	0.000	1.57–3.05
Visited health facility			
No (Ref)	1		
Yes	1.96***	0.001	1.31–2.92
Place of residence			
Rural (Ref)	1		
Urban	1.39	0.204	0.83–2.32
Region of residence			
Western	1		
Central	1.57	0.319	0.64–3.81
Greater Accra	1.22	0.630	0.54–2.78
Volta	0.47	0.149	0.17–1.31
Eastern	2.54	0.060	1.15–3.58
Ashanti	1.10	0.808	0.51–2.34
Brong Ahafo	1.18	0.722	0.47–2.93
Northern	0.13	0.062	0.04–1.45
Upper East	0.46	0.228	0.13–1.61
Upper West	0.76	0.608	0.27–2.16

Source: Computed from 2008 GDHS data set

*Ref* reference category

*p < 0.05; **p < 0.01; ***p < 0.001

The odds of contraceptive use among the working female adolescents were 2.99 times the odds of contraceptive use among their non-working counterparts. The odds were 2.89 times among those who had knowledge of their ovulatory cycle compared to their counterparts who had no knowledge of their ovulatory cycle. The odds were also 1.96 times among respondents who had ever visited any health facility compared to their counterparts who had not visited any health facility. As well, the odds of contraceptive use were 4.75 times among female adolescents who were married or living together compared to their counterparts who were not married.

## Discussion

This paper examined the prevalence and correlates of contraceptive use among female adolescents aged 15 to 19 in Ghana, using data from the 2008 Ghana demographic and health survey. The study found that older female adolescents were more than three times likely to practice contraceptive use than younger female adolescents. Perhaps, this is because older female adolescents are more mature and enlightened in terms of available contraceptive types and the importance of contraceptive use, compared to younger female adolescents who may be comparatively naive in terms of contraception. Besides, older female adolescents are more likely to be married, working and have obtained more education and more likely to be sexually active than their younger counterparts.

The likelihood of contraceptive use among the female adolescents increased significantly with the increase in level of education. Thus, educated female adolescents were more likely to use contraceptives than their uneducated counterparts. Likewise, Khan et al. [27] found a low contraceptive use among uneducated female adolescents in Bangladesh, while Tawiah [21] and Nketiah-Amponsah et al. [23] found level of education to be a significant predictor of contraceptive use among women in reproductive age in Ghana. This may result from the fact that educated women are more likely to be abreast of available contraceptives and are more likely to appreciate the positive impacts contraceptives have on their lives.

The study further found work status to be a significant correlate of contraceptive use among female adolescents. Female adolescents who were working were almost three times likely to use contraceptives than female adolescents who were not working. This is consistent with studies by Nketiah-Amponsah et al. [23], Khan et al. [27] and MacPhail et al. [28] which found that women who are employed are more likely to use any contraceptive method. The reason being that, working female adolescents may be adamant to keep their jobs and to have more time for their jobs instead of having babies, and with their ability to comparatively afford contraceptives compared to their non-working counterparts due to their working status, they are more likely to use any contraceptive than their non-working counterparts.

Knowledge of ovulatory cycle also had a significant relationship with female adolescents’ use of contraceptives. Female adolescents who knew their ovulatory cycle were more likely to use any contraceptive compared to their counterparts who did not know theirs. Perhaps, female adolescents who knew their ovulatory cycle may likely use several available contraception methods including the calendar or rhythm method of contraception, making them more likely to use contraceptives than their counterparts who did not know their ovulatory cycle. However, this could also be that women who use traditional methods of contraception may more likely know their ovulatory cycle.

It also came out that visit of health facility had a significant relationship with contraceptive use among female adolescents. Thus, female adolescents who visited health facilities were more likely to use any contraceptive than their counterparts who did not visit any health facility. This echoes what Khan et al. [27] found that female adolescents who were visited by family planning field workers were about two times more likely to use contraceptives than their counterparts who were not visited by family planning field workers. The implication is that visit to health facilities or being visited by health personnel is capable of improving contraceptive use among female adolescents and women in general. This may be as a result of the fact that more health facilities do render sexual and reproductive health services to women who visit such facilities. This may explain why female adolescents who visit health facilities are more likely to use contraceptives than their counterparts, even though women who want to or are using contraceptives may also likely visit a health facility.

Marital status also had significant relationship with contraceptive use among female adolescents. Consequently, contraceptive use was more likely among adolescents who were married or living together than among those who were not married. This confirms findings of a study conducted by Clemens and Madise [29] which found a significant relationship between marital status and contraceptive use in Ghana. On the contrary, studies including Nketiah-Amponsah et al. [23] and Okech et al. [30] found no significant relationship between marital status and use of contraceptives among females. More married female adolescents may likely use contraceptives because they may be more capable to afford contraceptives than their unmarried counterparts due to partner support. Hence, they were more likely to use contraceptives compared to their counterparts who were not married.

## Conclusions

Age, education, work status, knowledge of ovulation cycle, visit of health facility and marital status were the determinant factors for female adolescent contraceptive use in Ghana. Pragmatic adolescent and reproductive health programmes can therefore target younger female adolescents aged 17 and below at the Basic and Secondary schools as well as female adolescents without any formal education. This will help to promote contraceptive use among the younger ones and will encourage more uneducated female adolescents to use contraceptives in order to reduce teenage pregnancy and child birth.

Mothers or female guardians of female adolescents should be encouraged via the mass media to intensify the education of their female adolescents on their ovulatory or menstrual cycle early enough before menarche. ASRH services which are youth-friendly should also be made affordable and accessible to non-working female adolescents as well as the unmarried female adolescents. Education on the need for health facility use particularly among sexually active female adolescents should also be intensified by government, policy makers, decision makers and other stakeholders. This will not only increase health facility use and contraceptive prevalence among sexually active female adolescents in Ghana but will also reduce teenage pregnancy and child bearing, and in turn contribute to the achievement of the Millennium Development Goal 5 of improving maternal health.
